# Underuse of Epinephrine for the Treatment of Anaphylaxis in the Prehospital Setting

**DOI:** 10.1155/2022/5752970

**Published:** 2022-04-15

**Authors:** Fabrice Dami, Roxane Enggist, Denis Comte, Mathieu Pasquier

**Affiliations:** ^1^Department of Emergency Medicine, University Hospital Centre (CHUV), Faculty of Biology and Medicine, University of Lausanne, Lausanne, Switzerland; ^2^Emergency Medical Services, Dispatch Centre, State of Vaud (Fondation Urgences-Santé), Lausanne, Switzerland; ^3^Faculty of Biology and Medicine, University of Lausanne, Lausanne, Switzerland; ^4^Service of Immunology, Lausanne University Hospital, Faculty of Biology and Medicine, University of Lausanne, Lausanne, Switzerland

## Abstract

**Background:**

Anaphylaxis is a life-threatening reaction. Its key management is rapid diagnosis and prompt administration of intramuscular epinephrine. There are many barriers to epinephrine use.

**Objective:**

To assess the performance of dispatchers at suspecting anaphylaxis, proposing epinephrine treatment, helping find an epinephrine autoinjector (EAI) and using it.

**Methods:**

This is a retrospective study. Calls classified as “anaphylaxis” or “allergy” were included, and voice recordings were reviewed. Clinical, environmental, and operational variables were collected. Anaphylaxis was suspected if sudden dyspnoea, abdominal symptoms (vomiting, abdominal pain, or diarrhoea), dizziness, or loss of consciousness were present.

**Results:**

The dispatch handled 120,618 dispatch calls. Dispatchers suspected 611 (0.5%) cases of allergy. Among those, 437 (72%) were deemed consistent with anaphylaxis: 65 patients received epinephrine prior to the dispatcher's advice, and dispatchers proposed the use of an EAI to 141 patients (38%). An EAI was available in 45 situations. The proposition was accepted on 18 cases and performed in 16 cases. The median time from the EAI being in hand and the injection was 50 seconds.

**Conclusions:**

Trained dispatchers are able to suspect anaphylaxis, decide when to treat and provide guidance on using an EAI, although their performance can be improved. There is a need for easier access to EAIs in public places.

## 1. Introduction 

Anaphylaxis is a severe, life-threatening generalized or systemic hypersensitivity reaction. Its key management is rapid diagnosis and prompt administration of intramuscular epinephrine. The estimated lifetime prevalence of anaphylaxis is 1.6% in the United States and 0.3% in Europe [1]. The European incidence rate is 1.5–7.9 per 100,000 persons [2]. The reported U.S. case fatality rate for patients with anaphylaxis presenting to a hospital or emergency department ranges from 0.25% to 0.33% in the United States [3] and from 0.65% to 2% in the United Kingdom [4]. While fatal cases of anaphylaxis are unusual [5], studies show that delayed administration of epinephrine increases mortality risk [6]. Its underuse has also been previously reported [7]. Lack of availability among patients (they have a prescription but no epinephrine on them), lack of availability in schools and camps, lack of epinephrine use even when available (fear of harm or being unsure if epinephrine is necessary), and incorrect technique when using an epinephrine autoinjector (EAI) have been described as barriers to epinephrine use in the prehospital system [8]. Emergency medical service (EMS) dispatchers are often the first healthcare contact for patients with acute allergic reactions and could potentially play an important role in the early recognition, decision to treat, and coaching to use EAIs. Calls regarding suspicion of anaphylaxis may represent 0.5% of all the dispatch call volume [4]. As a delay in epinephrine administration is associated with poor outcome and because significant secondary effects of inappropriate intramuscular epinephrine are unusual, the benefits of administration far outweigh the risks [4].

The purpose of this study was to describe the performance of dispatchers at suspecting anaphylaxis, proposing epinephrine treatment, and guiding the caller to use an EAI before the arrival of EMS.

## 2. Methods

### 2.1. Study Design

This is a retrospective cohort study from February 2018 to December 2019 (23 months).

### 2.2. Setting and Population

The Lausanne Emergency Dispatch Centre receives and processes all calls regarding the dispatch of ambulances, ground prehospital emergency physicians, and helicopter emergency medical responses. Low-acuity cases requiring medical advice or a home visit by a general practitioner are treated by another dispatch centre. Dispatchers are paramedics or nurses with at least 5 years of field practice. They use criteria-based protocols, which allow some autonomy regarding their educational background. Anaphylaxis is suspected in the case of one of the following symptoms: sudden dyspnoea, abdominal symptoms (vomiting, abdominal pain, or diarrhoea), dizziness, or loss of consciousness, occurring after a possible trigger (food ingestion, insect bite, or medication), even if it is the first time. In this situation, a specific protocol is applied where dispatchers have to send an advanced life support team, help find a nearby EAI (patient's own, relative's, public or school one, or nearby pharmacy), and guide the caller to use it. Dispatchers have within their computer-aided system pictures and user guides for all the available EAIs on the market. As soon as an EAI is available, they ask which brand it is and then proceed to the prearrival instruction (injection) directly with the patient or with a bystander. If the EAI has expired or if the dosage available does not correspond to the patient's age or weight, dispatch protocol requires performing the injection anyway. The protocol has no contraindication, even in case of haemophilia, anticoagulant therapy, or cardiac condition as the benefit of epinephrine outweighs its risks through beneficial effects regarding a suspicion of anaphylaxis.

### 2.3. Data Collection

All calls were eligible. Calls classified as “anaphylaxis,” “allergy,” or containing one of those two words within the dispatcher's free text were included.

Voice recordings of included calls were reviewed by a medical student and/or one of the authors, an emergency physician (FD). The following variables were collected solely based on the audio tapes: key symptoms (state of consciousness, presence of dyspnoea, cutaneous-mucosal oedema, itching, and abdominal symptoms), suspected trigger, known history of allergy, medication received prior to the call, availability of an EAI, proposition to use the EAI by the dispatcher, acceptance of EAI use by the patient or bystander, location of incident, time from decision to injection, and problem(s) with the injection.

The study team checked if dispatchers asked all the necessary questions to evaluate the presence of the possibility of anaphylaxis. The team also ruled out whether the call was a case of simple allergy (cutaneous-mucosal oedema or itching) or consistent with anaphylaxis according to the presence of one of the following criteria: dyspnoea, dizziness, loss of consciousness, or gastrointestinal symptoms.

Paramedics on-site documented the severity of cases using the National Advisory Committee for Aeronautics (NACA) score (Supplementary File 1), which was collected for all cases.

### 2.4. Ethical Authorization

The local ethical committee exempted this work from the need to submit a formal demand as access to the paramedics or hospital charts was not solicited; therefore, it was not under the scope of human research law because no vitals were collected (req 2020–00230).

### 2.5. Data Analysis

Demographic and clinical characteristics are summarized using descriptive statistics, including medians and interquartile ranges for continuous variables and frequencies for categorical variables. All data were entered into a Microsoft Excel spreadsheet (Microsoft Corp., Redmond, Washington, USA).

## 3. Results

### 3.1. Calls and Patient Characteristics

During the study period, the dispatch centre handled 120618 calls. Dispatchers suspected 611 (0.5%) cases of allergy. The median age of patients was 66 (IQR 40–82) years. There was one cardiac arrest with return of spontaneous circulation and 56 life-threatening situations according to the NACA score documented by paramedics (NACA > 4). The median time for EMS arrival on-site was 15 (IQR 8–17) minutes. A total of 125 patients (20.5%) were not transported, of whom none benefited from epinephrine use guided by dispatch or self-use.

Among these 611 patients suspected of allergy, mucocutaneous symptoms were the most frequently described (*n* = 411, 67%), followed by dyspnoea (*n* = 328, 54%) and gastrointestinal symptoms (*n* = 104, 17%). The most common suspected triggers identified by the caller were food (*n* = 181, 30%), insect bites (*n* = 132, 22%), medications (*n* = 94,15%), and radiocontrast agents (*n* = 13, 2%). The most frequent place of occurrence of an allergic reaction was a private place (*n* = 317, 52%), followed by public places (mainly schools and sports facilities, including outdoor swimming pools) (*n* = 170, 28%) and medical facilities (GP offices and radiology practices) (*n* = 80, 13%). The medications administered before the call were H1-antihistamines (*n* = 108, 18%), glucocorticoids (*n* = 38, 6%), inhaled salbutamol (*n* = 13,2%), and epinephrine (*n* = 99, 16%) (Table 1).

### 3.2. Anaphylaxis Determination

Among the 611 calls of suspected allergy, 437 (72%) cases were deemed consistent with anaphylaxis after the call was reviewed.  Table 2 provides the interview performance in those 437 cases; questions regarding abdominal symptoms were not asked on 133 occasions (30.4%).

Among the 174 cases considered as immediate allergy without symptoms of anaphylaxis, 34 cases were not thoroughly evaluated for anaphylaxis by dispatchers (incomplete interview).

### 3.3. Availability of Epinephrine and Prearrival Instructions

**Figure 1:**
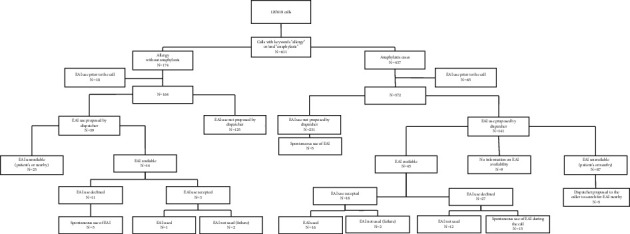
Flowchart.

There were 437 cases of suspected anaphylaxis, including 65 patients who received epinephrine prior to the dispatcher's advice. Among the 372 patients left, dispatchers proposed the use of an EAI to 141 patients (38%), and an EAI was available in 45 situations in this group. The proposition was accepted in 18 cases and performed in 16 cases (two failures) (Table 3). Within the anaphylaxis group, excluding EAI use prior to contacting a dispatcher, prearrival instructions were initially declined on 27 occasions but were ultimately performed spontaneously on 15 occasions (Figure 1). The reasons for declining the use of an EAI were fear/anxiety in the patient or bystander, prompt EMS arrival, and refusal from the general practitioner on-site (fear and/or judging treatment unnecessary).

An EAI has also been used on simple allergy cases, either spontaneously by a patient or bystander (15 cases) or by a dispatcher's proposition (three cases, with only one being successful).

When successful, the median time from the EAI being in hand and the injection was 50 seconds, including one case without anaphylaxis (*n* = 17) (Table 3, Figure 1).

## 4. Discussion

Trained dispatchers are able to suspect anaphylaxis, decide when to treat, and provide guidance on using an EAI.

The definition of anaphylaxis is well described. However, in the context of dispatch, it needs to be adapted as clinical exam of the skin in particular cannot be performed in the absence of video and as knowledge of the classical triggers in a given setting, important to diagnose anaphylaxis, is often unknown [9]. Nevertheless, using the criteria described above, dispatchers were able to correctly suspect anaphylaxis in 437 calls among a total of 611 calls for allergy. When managing a call for a possible allergic reaction, asking for clinical signs and symptoms of anaphylaxis should be mandatory, especially regarding gastrointestinal symptoms, which are often overlooked by dispatchers. The study design, however, did not allow us to quantify how many cases of anaphylaxis were eventually missed.

In addition, when anaphylaxis was suspected, dispatchers should have systematically addressed the availability of a nearby EAI. If the patient did not have one, dispatchers must propose to use someone else's EAI, one from the school or workplace infirmary, or even one from a nearby pharmacy. In this study, dispatchers proposed to use an EAI in 141 situations among 372 (38%) suspected cases of anaphylaxis not having received epinephrine prior to the call, which is insufficient.

As for dispatch-assisted cardiopulmonary resuscitation (CPR) for suspected cardiac arrest [10], when dealing with suspicion of anaphylaxis, dispatchers should systematically propose the administration of an EAI. This may of course lead to a false positive, as shown in this work. But regarding the balance between the benefits and risks of inappropriate injection of intramuscular epinephrine, dispatchers should be taught to favour overtreatment in case of doubt. A parallel can be made with dispatch-assisted CPR, where the best dispatch centres may reach a false positive rate of 20% when proposing telephone CPR but then leave almost no case of cardiac arrest without telephone CPR.

Scant data are currently available on the role of emergency call centre dispatchers in the management of patients with anaphylaxis. A recent American study, in which emergency call voice recordings were reviewed, noted that EMS dispatchers did not systematically adhere to medical guidelines to identify anaphylaxis and infrequently inquired about the availability of epinephrine or informed callers about its use, which is something also shown in this work. The authors emphasized the importance of education and optimal protocol design to increase the recognition of anaphylaxis by emergency call centre dispatchers [11].

Some argue there is a need for broader awareness in society about the symptoms and treatment of anaphylaxis to improve epinephrine use, as it has been developed for cardiac arrest and automated external defibrillator use [7]. However, given a much lower incidence of anaphylaxis (2–8/100,000 population) [1]compared to cardiac arrest (50–100/100,000 population) [12], it may seem more efficient to focus education efforts on emergency call dispatchers only rather than training an entire population. Since the first episode of anaphylaxis may occur without a history of allergy and because patients may not carry their EAI, it is essential to obtain prompt instruction on the availability of a nearby EAI and how to use it. Therefore, the education of dispatchers is probably the best way to achieve this task.

In this study, patients, bystanders, and even physicians were sometimes reluctant to inject epinephrine in cases of anaphylaxis favouring H1-antihistamine drugs, which is concerning that they may reduce the likelihood or progression to anaphylaxis but are not frontline treatment [13].Training dispatchers may improve the delivery of epinephrine by convincing doubtful bystanders of its prompt administration.

This study also demonstrated that schools are the most common public place where anaphylaxis occurs. The EpiPen4Schools pilot survey from 2012 revealed that 10% of over 6000 participating schools in the United States reported an anaphylactic event—22% of these patients had no history of allergy and 25% were not treated by epinephrine [14]. The authors concluded that there is a need for schools to stock EAIs. Stocking EAIs is one thing, but there is also a growing concern about preparing schools to recognize and treat anaphylaxis [15]. The EpiPen4Schools survey from 2013 demonstrated that among 55% of the 6000 participating schools, only a nurse or selected staff were authorized to administer epinephrine [16]. A French study reported the same difficulty of access to EAIs within schools [17]. There is an argument to equip schools with unassigned, accessible, and unlocked EAIs and allow emergency call centre dispatchers to decide when to treat and guide users, even in the absence of nurses or trained staff on-site. This could reduce the burden of having nurses on-site during open hours (availability and costs).

In the United States, the Federal School Access to Emergency Epinephrine Act was enacted in 2013. This legislation encourages states to adopt laws that encourage school to stock EAIs. In the state of New York, the Emergency Allergy Treatment Act authorizes (not obliges) public venues, such as restaurants, youth organizations, sports leagues, theme parks, port arenas, and daycare and educational facilities to stock and administer EAIs in case of an emergency to individuals who seem to have anaphylactic symptoms. These political movements tend toward easier access to life-saving treatments. A liberal system of distribution of EAIs without medical prescription for public places (restaurants, outdoor areas, and schools) is necessary. For example, this could be coupled with AEDs in some places.

Based on public access defibrillation programmes, some communities have deployed EAIs in their territory to facilitate access to epinephrine. For example, in Canada, where a medical prescription is not necessary to obtain an EAI, communities, schools, or sports venues are able to equip themselves with such devices [18, 19]. The requirement for a medical prescription can be an important barrier for public access to EAIs.

All these obstacles must be overcome to improve the early use of epinephrine in the prehospital environment. The education of emergency call dispatchers on how to recognize and manage anaphylaxis adequately goes in the same direction. As the benefit of intramuscular epinephrine treatment far outweighs the risk of side effects, the decision to treat can be delegated to emergency call dispatchers within specific protocols.

### 4.1. Limitations

This is a retrospective monocentric descriptive study. Only cases tagged or containing the keywords “allergy” or “anaphylaxis” in the files of dispatchers were included. Therefore, the undertriage of allergies by dispatchers could not be assessed. Similarly, if information on the quality of breathing, state of consciousness, and presence or absence of abdominal symptoms were missing among the included cases of allergy, some cases of anaphylaxis may have been missed.

Calls classified as anaphylaxis are suspicions and not definitive diagnoses. Also, the definition of anaphylaxis used by dispatchers is different from the clinical one, as no clinical status is feasible. Therefore, regarding skin symptoms which are not included in the diagnostic criteria in this study, it may cause some undertriage. Telemedicine may reduce this shortcoming in the near future.

As this study takes into account calls from a sole dispatch centre that only takes acute care calls, it does not provide an accurate incidence rate of allergies within the population.

## 5. Conclusion

Trained dispatchers are able to suspect anaphylaxis, decide when to treat, and provide guidance on using an EAI, although their performance can be improved. In the meantime, it is necessary to improve the availability of EAIs in public places, such as schools, restaurants, outdoor recreation areas, and other venues. By easing the possibility of acquiring or accessing an EAI, public authorities, in addition to improving training within dispatch centres, may contribute to fill the gap for better access to epinephrine.

## Figures and Tables

**Table 1:** Characteristics of calls.

	Total calls (*n* = 611)	Anaphylaxis (*n* = 437)
Patient's age (years)		
0–20	159	120
21–30	81	57
31–65	286	204
>65	61	40
Unknown	24	16

Symptoms		
Unconsciousness/dizziness	36	36
Dyspnoea	328	328
Mucocutaneous	411	279
Gastrointestinal	104	104
Prior allergy history	269	195
Suspected trigger identified by caller	447	318
Food	181	132
Nuts	27	18
Peanuts	14	14
Eggs	12	9
Shellfish	11	8
Fish	6	5
Sesame	5	3
Almonds	5	4
Fruits	19	12
Dairy products	8	6
Unknown	74	53
Stinging insect	132	76
Medication	94	65
Antibiotics	26	18
Unknown	68	47
Radiocontrast agents	13	10
Desensitization	10	10
Cosmetics	8	8
Pollen	4	2
Physical exercise	2	1
Others	3	4

Location of incident		
Private location	317	219
Home	311	213
Car	6	6
Public location	170	112
School	41	33
Pharmacy	15	10
Nursery	9	6
Sports facilities	14	11
Restaurant	10	6
Hotel	9	6
Street	9	4
Swimming pool	5	1
Camping	4	2
Jail	2	2
Park	9	1
Store	5	0
Others	38	30
Medical facilities (GP office or radiology practice)	80	54
Workplace	35	28

Medication prior to the call		
IM epinephrine	75	65
H1-antihistamine	80	21
Oral steroids	36	5
Inhaled beta-2 agonist	8	3
Availability of epinephrine (including self-use)	134	110
Patient's own	59	49
Medical office	18	16
School	6	6
Someone else	6	4
Pharmacy	0	2
Unknown	45	33

**Table 2:** Performance of dispatchers' interviews for calls consistent with anaphylaxis (*N* = 437).

	Yes	No	Unknown
Questions asked			
Is patient conscious?	390	36	11
Is patient breathing normally?	73	328	36
Does patient have abdominal symptoms?	279	25	133

**Table 3:** Epinephrine proposition and administration with respect to anaphylaxis determination.

	Allergy consistent with anaphylaxis (*n* = 437)	Allergy inconsistent with anaphylaxis (*n* = 174)
Epinephrine administered prior to call	65 (14.9%)	10 (5.7%)
Epinephrine administered during the call without proposition by the dispatcher	5 (1.1%)	0 (0%)
Epinephrine proposed by dispatcher (cases not having received epinephrine)	141 (37.9%)	39 (23.8%)
Epinephrine use accepted following dispatcher's proposal	18 (4.1%)	3 (1.7%)
Failure to administer epinephrine following dispatcher's proposal	2 (0.5%)	2 (1.1%)

## Data Availability

The data used to support the findings of this study are available from the corresponding author upon request.

## Abbreviations

EAI: Epinephrine autoinjector
EMS: Emergency medical services.

## Additional Points

*Key Messages*. Dispatchers can contribute to improve epinephrine treatment for anaphylaxis prior to the arrival of emergency medical services. There is a lack of public/school availability of epinephrine autoinjectors. *Capsule Summary*. Improving the performance of dispatchers at recognizing and treating anaphylaxis, associated with public health regulation easing access to epinephrine autoinjectors, may improve early treatment of anaphylaxis.

## Supplementary Materials

Supplementary Materials Supplementary file 1: NACA score: the National Advisory Committee for Aeronautics (NACA) score is used by many emergency medical services to assess the severity of prehospital patients.
