# Supplementary material for: Underuse of Epinephrine for the Treatment of Anaphylaxis in the Prehospital Setting
Source: Emerg Med Int. 2022 Apr 15;2022:5752970. doi: 10.1155/2022/5752970 (PMC9033371; doi:10.1155/2022/5752970)
Supplement: Supplementary Materials — Supplementary file 1: NACA score: the National Advisory Committee for Aeronautics (NACA) score is used by many emergency medical services to assess the severity of prehospital patients. [file 5752970.f1.docx]

***National Advisory Committee for Aeronautics (NACA) score revised by the State of Vaud (2005)***

*NACA 0 No injury or disease*

*NACA 1 Injuries/diseases without any need for acute physicians care*

*NACA 2 Injuries/diseases requiring examination and therapy by a physician but hospital admission is not indicated. Including: large contusions, finger and toe fracture, 2^nd^ degree burn (10-20% of body surface), exhaustion without hypothermia*

*NACA 3 Injuries/diseases without acute threat to life but requiring hospital admission. Including: maxillofacial trauma, wound with vascular/neurological impact, 3^rd^ degree burn (10-20%), hypoglycemia without coma, TIA, supra-ventricular arrhythmia with conserved hemodynamic, right iliac fossa pain syndrome, hypothermia stage I, 2^nd^ degree burn (20-30%), isolated limb fracture (femur excluded)*

*NACA 4 Injuries/diseases which can possibly lead to deterioration of vital signs. Including: open skull fracture, hypothermia stage II, suspicion SCA, suspicion ectopic pregnancy/placenta praevia*

*NACA 5 Injuries/diseases with acute threat to life. Including: head trauma GCS<8, heart infarct, bradycardia (< 30/min), tachycardia (>180/min), complete heart bloc, eclampsia, hypothermia stage III, haemodynamic shock, multiple ribs fractures, acute dyspnea, pulmonary edema*

*NACA 6 Injuries/diseases transported after successful resuscitation. Including: chest trauma with severe dyspnea, aortic rupture, airways total obstruction, central apnea, emergency external pacing, cardiac arrest (ventricular fibrillation or asystole from any cause)*

*NACA 7 Lethal injuries or diseases (with or without resuscitation attempts)*
